# Controllable Synthesis of Titanium Silicon Molecular Zeolite Nanosheet with Short b-Axis Thickness and Application in Oxidative Desulfurization

**DOI:** 10.3390/nano14110953

**Published:** 2024-05-29

**Authors:** Tieqiang Ren, Yujia Wang, Lulu Wang, Lisheng Liang, Xianming Kong, Haiyan Wang

**Affiliations:** 1 College of Chemistry and Chemical Engineering, China University of Petroleum (East China), Qingdao 266580, China; tqr_lnpu_121@126.com (T.R.); fsyzww@126.com (Y.W.); gracewangl@163.com (L.W.); 2 School of Petrochemical Engineering, Liaoning Petrochemical University, Fushun 113001, China; 3 Petroleum Engineering Research Institute, Petrochina Dagang Oil Field Company, Tianjin 300280, China; 13312041240@163.com

**Keywords:** titanium silicon molecular zeolite, PDDA, nanosheets, tunable short b-axis thickness, desulfurization

## Abstract

Titanium silicon molecular zeolite (TS-1) plays an important role in catalytic reactions due to its unique nanostructure. The straight channel on TS-1 was parallel to the orientation of the short b-axis and directly exposed to the aperture of the 10-member ring with a diameter of 0.54 nm × 0.56 nm. This structure could effectively reduce the diffuse restriction of bulk organic compounds during the oxidative desulfurization process. As a kind of cationic polymer electrolyte, polydimethyldiallyl ammonium chloride (PDDA) contains continuous [C_8_H_16_N^+^Cl^−^] chain segments, in which the Cl^−^ could function as an effective structure-directing agent in the synthesis of nanomaterials. The chain of PDDA could adequately interact with the [0 1 0] plane in the preparation process of zeolite, and then the TS-1 nanosheet with short b-axis thickness (6 nm) could be obtained. The pore structure of the TS-1 nanosheet is controlled by regulating the content of PDDA. Scanning electron microscopy (SEM), transmission electron microscopy (TEM), X-ray photoelectron spectroscopy (XPS), N_2_ physical adsorption analysis, infrared absorption spectrum and ultraviolet–visible spectrum were used to determine the TS-1. The thinner nanosheets exhibit excellent catalytic performance in oxidative desulfurization of dibenzothiophene (DBT), in which the removal rate could remain at 100% after three recycles. Here, the TS-1 nanosheet with short b-axis thickness has a promising future in catalytic reactions.

## 1. Introduction

Fine desulfurization of fossil fuels has attracted considerable interest as an environmental pollution problem [1,2]. There are a lot of desulfurization methods, including oxidative desulfurization (ODS), which is an effective, high-efficiency technology used to obtain fuels with lower sulfur content under mild conditions [3]. TS-1 was first reported in 1983 [4]. Numerous research studies of TS-1 were developed in selective oxidation and epoxidation reactions of organic compounds, in which hydrogen peroxide (H_2_O_2_) and ter-butyl hydroperoxide (TBHP) were usually applied as oxidants. As an environmentally friendly feature, H_2_O_2_ is usually employed in catalytic reactions under mild reaction conditions [5].

TS-1 has been extensively applied for manufacturing oxygenates, in which H_2_O_2_ is usually employed as an oxidizing organic reactant, such as in the hydroxylation of benzene, epoxidation of olefins, and ammoxidation of cyclohexanone [6,7]. However, the small size (0.55 nm) of the micropore and framework structure in TS-1 zeolite hinders the diffusion of organic sulfur, especially for the molecules that are adsorbed on Ti-active centers. The microspore property of TS-1 further constrains the mass transfer of sulfides, which limits its application in oxidative desulfurization [8,9]. The catalytic oxidation performance of TS-1 is highly dependent on the content of Ti and the accessibility of the target in the active site of the catalyst.

The hierarchical TS-1 has attained considerable attention as an additional mesoporous pore formed between intercrystalline and interconnected intracrystalline, and is beneficial for mass transfer, which is a feature that could improve the efficiency of catalytic reactions [10,11]. The hierarchical structure in TS-1 zeolite could provide a mass transfer route and enhance the accessibility of the active sites in the TS-1 framework. Several methods [12,13,14,15,16] have been proposed for the preparation of mesoporous zeolites with hierarchical structures, such as the partial crystallization of amorphous mesoporous structure [17], zeolite seeds assisting in building primary structure units [18], the crystallization of mesocellular silica foam into a hierarchical structure [19], and a hydrothermal approach using a special surfactant [20]. As a kind of surfactant molecule, the cationic polymers can electrostatically adsorb onto nanocrystals and generate zeolite nanoparticles with hierarchical structure through a self-assembly process [21,22]. The spherical micelle, cylindrical micelle, and lamellar micelle could be formed through the self-assembly of surfactant molecules that could serve as a template for producing the mesopore structures of zeolite [23,24,25]. These nanoscopic micelle structures could incorporate meso-macropores into microporous zeolites with substantial structures [26,27].

Some researchers have confirmed that the single-crystalline zeolite beta with inter-connected mesopores could be synthesized by using a commercial polymer, PDDA, as a dual-functional template [28]. The single-crystalline nature endowed Beta-MS with better hydrothermal stability and exhibited remarkably higher catalytic activity compared with surfactant-derived mesoporous zeolite Beta. PDDA has also been successfully applied in the regulation of hierarchical TS-1 zeolites with abundant intracrystalline mesopores and regular hexagonal morphology [29]. However, the manufacture of lamellar material with a hierarchical structure has not been reported, and the PDDA content should be a crucial factor.

In this work, a PDDA-assisted synthetic strategy was developed for constructing a hierarchical TS-1 stacked nanosheet with tunable short b-axis thickness, in which the PDDA mainly served as a mesopore template and structure-directing agent. TS-1 zeolite was successfully synthesized through a PDDA electrostatic adsorption route under conventional hydrothermal process, and the TS-1 nanosheets (PTS) showed superior catalytic performance compared with conventional TS-1 zeolite in the oxidative desulfurization of thiophenic sulfur [30].

## 2. Materials and Methods

### 2.1. Reagents and Chemicals

Tetraethyl orthosilicate (TEOS, 98%, Shanghai Macklin Biochemical Co., Ltd., Shanghai, China), tetrapropylammonium hydroxide (TPAOH, 25%, Beijing InnoChem Science & Technology Co., Ltd., Beijing, China), tetrabutyl titanate (TBOT, 98%, Guangfu Fine Chemical Research Institute, Tianjin, China), polydiallyldimethyl ammonium chloride (PDDA, Mw 100,000~200,000, 20%, Shanghai Yuanye Bio-Technology Co., Ltd., Shanghai, China), noctane (95%, Shanghai Macklin Biochemical Co., Ltd.), hydrogen peroxide solution (H_2_O_2_, 30%, Sigma-Aldrich chemicals Co., Ltd., St. Louis, MO, USA), dibenzothiophene (DBT, 98%, TCI Shanghai Development Co., Ltd., Shanghai, China), and ethanol (100%, Tianjin Wind Boat Chemical Reagent Technology Co., Ltd., Tianjin, China) were used in this experiment.

### 2.2. Synthesis of Conventional TS-1(CTS)

Conventional TS-1 zeolite was prepared using a hydrothermal method in which a mixture of SiO_2_/TiO_2_/TPAOH/H_2_O/EtOH with a molar ratio of 30/1/12/2100/120 was used as a starting gel. Typically, 5.1915 g TPAOH (25%) was mixed with deionized water under violent stirring for 30 min and then 3.32 g TEOS was added dropwise under stirring for another hour. Finally, the mixture of 0.181 g TBOT and 2.94 g ethanol was dispersed into the above solution under continuous stirring for 2 h. The resultant gel was transferred into a Teflon-lined stainless-steel autoclave. After crystallization at 150 °C for 48 h, the solid product was centrifuged twice, rinsed thoroughly with deionized water, and dried overnight at 80 °C. Then, the product was calcined at 550 °C for 8 h in air condition, and the conventional TS-1 (CTS) was obtained.

### 2.3. Synthesis of TS-1 Nanosheets (PTS)

The starting gel of SiO_2_/TiO_2_/TPAOH/H_2_O/EtOH with a molar ratio of 30/1/12/2100/120 was prepared according to the above procedure, and then PDDA was dispersed in the starting gel under stirring for 1 h. The content of PDDA in the starting gel was set at x wt %, where x is 0.010, 0.015, 0.030, 0.090, and 0.180, respectively. Following the above procedure of crystallization and calcination, the TS-1 nanosheets with ultra-thin lamellar structure were obtained and named x PTS.

### 2.4. Characterizations

The X-ray diffraction (XRD) spectra were obtained with a Bruker D8 Advance diffractometer (Bruker, Billerica, MA, USA) equipped with Cu Kα (λ = 1.5418 Å, 40 kV, 40 mA). Transmission electron microscopy (TEM) images and selected area electron diffraction (SAED) patterns were observed on a JEM 2100F electron microscope (JEOL Ltd., Tokyo, Japan) with an accelerating voltage of 200 kV. Infrared (FT-IR) spectra were collected using a Nicolet iS50 infrared instrument (Nicolet Instrument Corporation, Madison, WI, USA) with a KBr compression method in the range of 400 cm^−1^ to 4000 cm^−1^. The UV–vis diffuse reflectance spectra (DRS) were recorded in the range between 200 nm and 400 nm with an Agilent Cary5000 spectrophotometer (Agilent, Santa Clara, CA, USA). N_2_ adsorption–desorption experiments were performed with an automated gas sorption analyzer of Quantachrome autosorb iQ_2_. XPS spectroscopy (ESCALAB 250Xi+, Thermo Fisher Scientific, Waltham, MA, USA) was employed to obtain the core-level information of zeolite under the test condition of passing energy of 20 eV, residence time of 50 ms, step length of 0.05 eV, and 20 runs. The content of sulfur was measured with a TSN5000 sulfur analyzer (Jiangsu Electric Analysis Instrument, Jiangsu Skyray Instrument, Kunshan, China).

### 2.5. Catalytic Performance Test

The solution of noctane with DBT was used as the model fuel, in which the concentration of sulfur was 500 ppm. The oxidative desulfurization reaction was performed in a conical flask (50 mL) reactor at 60 °C and equipped with an air condenser tube. In a typical reaction, 20 mL of model fuel, 15 mL of H_2_O, 0.05 mL of H_2_O_2_, and 0.15 g of a catalyst were added into the reactor. The oxidative desulfurization reaction was carried out at 60 °C for 105 min. The upper oil phase was extracted at a 15 min interval. After that, the concentration of organic sulfur in the oil phase was measured and the removal rate (%) was calculated based on the results.

## 3. Results and Discussion

### 3.1. Morphology and Characterization of CTS and PTS Zeolites

**Figure 1:**
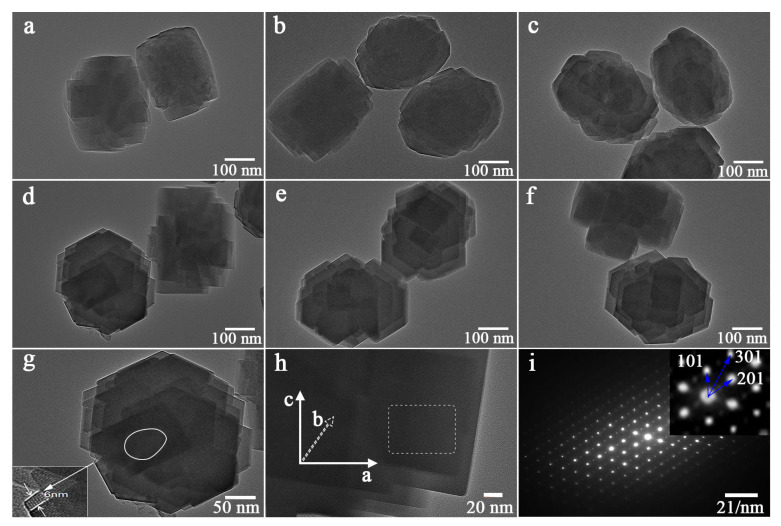
TEM images of CTS and PTS prepared with different amounts of PDDA: (**a**) CTS. (**b**) 0.010PTS. (**c**) 0.015PTS. (**d**) 0.030PTS. (**e**) 0.090PTS. (**f**) 0.180PTS. (**g**,**h**) High-magnification TEM for 0.030PTS. (**i**) The SAED pattern of selected region in (**h**) for 0.030PTS.

The TEM images of the TS-1 zeolites are shown in Figure 1. The bright gray areas in the images were due to the uniform micropores and hierarchical mesopores inside the zeolites. There is an obvious difference between the morphologies of zeolites prepared with different conditions. The bright gray regions with an irregular distribution of hierarchical pore structures of TS-1 zeolites were observed (Figure 1a–c). In contrast, the continuous mesopores were observed from the 0.030PTS, 0.090PTS, and 0.180PTS zeolites presented in the regular bright gray regions (Figure 1d–f). The high-magnification TEM image of 0.030PTS is shown in Figure 1g, in which the ultra-thin lamella structure (6 nm) was observed and continuous mesopores were marked with white lines. As shown in the SAED pattern (Figure 1h,i), the 0.030PTS zeolite showed single-crystalline properties and was presented in the distinguishable [0 1 0] plate. Three lattice planes of [1 0 1], [2 0 1], and [3 0 1] were observed from the 0.030PTS, and the straight channels of microporous structures were well exposed in 0.030PTS crystal along the short b-axis orientation. Based on the TEM images and SAED pattern, it could be concluded that PTS mesocrystals of nanosheets have an orderly stacked structure. The exposed [0 1 0] plates, ultra-thin lamellas (6 nm), and textural properties (Table 1) were beneficial for the oxidation desulfuration.

**Figure 2:**
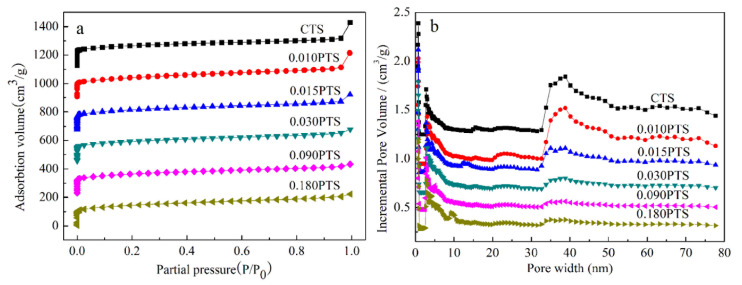
Nitrogen adsorption–desorption analysis of CTS and PTS zeolites: (**a**) Adsorption–desorption isotherm. (**b**) Pore size distribution of cylindrical pore model using DFT method.

The N_2_ adsorption–desorption isotherms of CTS and PTS zeolites are shown in Figure 2a. All samples presented a typical I adsorption isotherm based on the IUPAC classification, which indicated that the TS-1 zeolites have a typical microporous structure. The pore distribution of the cylindrical pore model for TS-1 zeolites was calculated using the density functional theory (DFT) method as shown in Figure 2b, in which the intrinsic micropores, hierarchical mesopores, and continuous mesopores were obtained. The micropore volumes of all zeolites ranged from 0.1722 to 0.1914 cm^3^·g^−1^ (Table 1, e), and the difference in micropore volumes was caused by the intercrystalline microchannel continuity. These results indicated that the intrinsic micropore of the MFI structure was almost unchanged with the different dosages of PDDA used in the starting gel.

The mesoporous and microporous surface areas of CTS zeolite were 85.11 m^2^·g^−1^ and 423.9 m^2^·g^−1^, respectively. In contrast, the surface areas of 0.010PTS zeolite were 139.5 m^2^·g^−1^ and 368.6 m^2^·g^−1^, as shown in (Table 1, b,c). The variation of the surface areas of 0.010PTS was due to the strong interaction between the polymer chain of PDDA and the growing crystal for zeolite, in which the PDDA functioned as a template agent for generating more mesoporous volume (Table 1, f). However, the external specific surface and mesoporous volume were decreased as the dosage of PDDA was further increased in the starting gel. As result, the 0.030PTS zeolite had a relatively high external specific surface and the lowest mesoporous volume. Obviously, The PDDA could generate more external surfaces, and the [0 1 0] plates of PTS zeolites were even exposed (Figure 1i). It was concluded that the PDDA was most likely adsorbed on the [0 1 0] plate and restricted the growth of crystals along the b-axis orientation.

**Figure 3:**
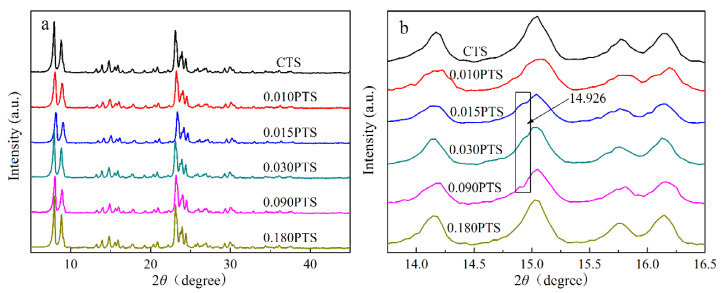
XPD powder patterns: (**a**) XRD characterization of CTS and PTS zeolites. (**b**) The [3 0 1] crystal plate of 0.030PTS zeolite.

The XRD patterns of CTS and PTS zeolites were measured and shown in Figure 3. Five diffraction peaks were observed at 7.9°, 8.8°, 23.1°, and 23.9°, which were assigned to the typical MFI structure of TS-1 zeolites (PDF#01-070-6302) [30,31]. There was no diffraction peak observed at 25.4°, which indicated the absence of the TiO_2_ anatase phase. Furthermore, no additional peaks were observed, which confirmed the high phase purity of the zeolites. The diffraction peaks of CTS, 0.010PTS, and 0.180PTS zeolites showed higher intensity than the other samples because they had bigger crystal sizes of 40.8 nm, 37.0 nm, and 36.8 nm, as calculated by the Scherrer formula. Meanwhile, the other three zeolites had proximate and lower diffraction intensity, which indicated that the smaller crystal grains were formed. The 0.030PTS zeolite only showed a crystal size of 27.3 nm. It is noteworthy that the weak diffraction peaks of the [3 0 1] crystal plane could be observed at 14.926° from 0.015PTS, 0.030PTS, and 0.090PTS zeolites (Figure 3b). This had undoubtedly been observed in the TEM images (Figure 1i). The species adsorbed by PDDA between adjacent nanosheets could bring continuous mesoporous channels, accompanied by the changes in TS-1 zeolite aperture distributions (Figure 2b).

**Figure 4:**
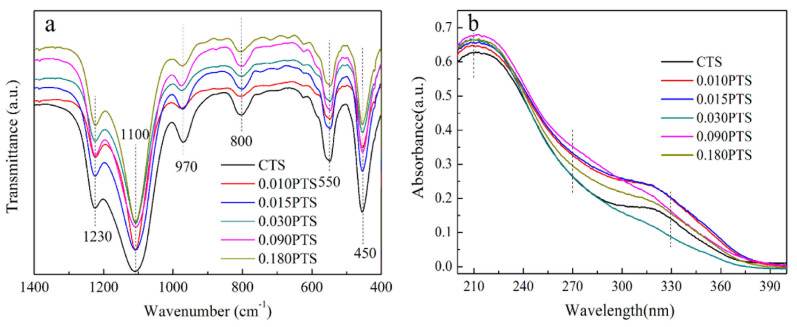
Structure composition analysis: (**a**) FT-IR spectra. (**b**) UV–vis spectra.

FT-IR spectra was used to determine the surface groups of TS-1 zeolites (Figure 4a), and six obvious peaks were observed at 450 cm^−1^, 550 cm^−1^, 800 cm^−1^, 970 cm^−1^, 1100 cm^−1^, and 1230 cm^−1^. The peaks at 550 cm^−1^, 800 cm^−1^, and 1230 cm^−1^ belonged to the stretching vibrations of the -O-Si-O- and -O-Ti-O- groups from the double five-membered rings [32]. The peak at 970 cm^−1^ was attributed to the stretching vibration of the Si-O-Ti bond and Si-O bond that was perturbed by the framework titanium species [33,34]. The intensity ratio of I970/I800 was commonly used to evaluate the relative content of Ti species in the framework structure, which was 1.18, 1.09, 1.14, 1.10, 1.13, and 1.12 corresponding to CTS, 0.010PTS, 0.015PTS, 0.030PTS, 0.090PTS, and 0.180PTS, respectively. The intensity ratios of I970/I800 indicated that the relative content of Ti in the framework structure was decreased by adding PDDA into the precursor gel. Meanwhile, it was also accompanied by the increment of surface Ti species content (Table 2, b).

The UV–vis DRS spectra were used to investigate the chemical environment of titanium species in TS-1 (Figure 4b). There are two major peaks presented at 210 nm and 330 nm. The strong peak at 210 nm was assigned to the charge transfer between titanium and oxygen atoms in tetrahedral coordination [8]. This result verified that the titanium atoms entered the framework of TS-1 zeolites. The weak peak around 270 nm corresponded to the partially polymerized titanium species with hexagonal coordination [35], and the absorption peak around 330 nm confirmed the existence of anatase TiO_2_ in TS-1 zeolite. However, there was no characteristic peak in the anatase phase of TiO_2_ observed in the XRD powder patterns due to a smaller amount of incorporation or the small particle size of the anatase phase [36].

**Figure 5:**
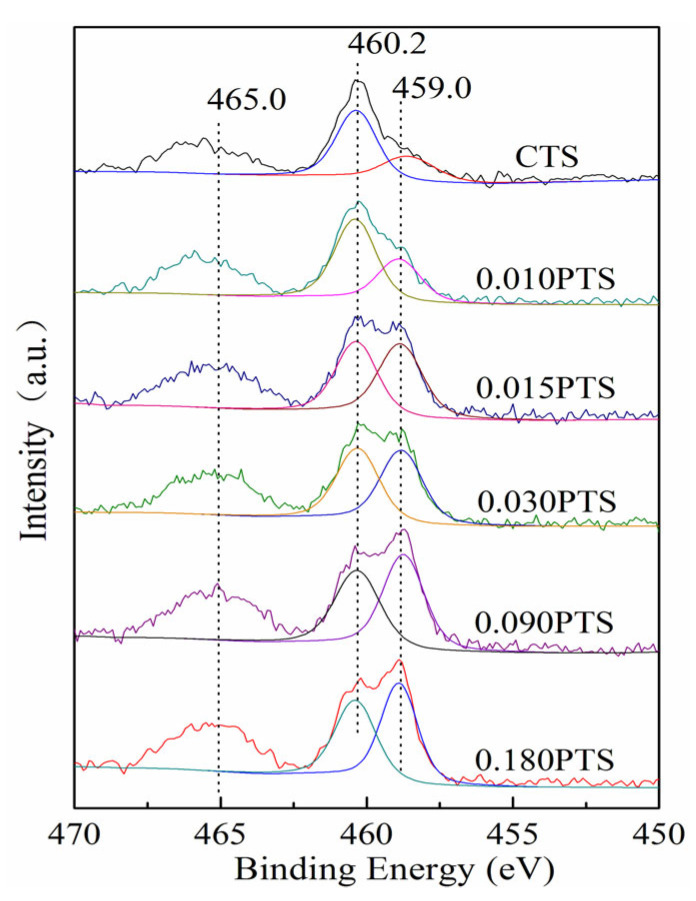
Ti 2p region for CTS and PTS zeolites.

XPS spectroscopy was employed to investigate the content and the chemical state of the Ti species on the external surface of zeolites. The binding energy for carbon at 284.8 eV was used to make binding energy correction. The chemical state was observed after peak fit analysis of XPS spectra, as shown in Figure 5. The Ti 2p_3/2_ at 460.2 eV and Ti 2p_1/2_ at 465.0 eV were assigned to the framework Ti species in tetrahedral coordination. As the increment of PDDA, the Ti 2p_3/2_ at 460.2 eV split off a new band at 459.0 eV and became narrower, indicating the existence of new Ti species in hexahedral coordination (TiO_6_) [37]. The oxidation mechanisms over TS-1 were investigated by means of DFT/ONIOM_2_ calculations [38]. The calculated activation energy of oxidation with TS-1 containing non-framework Ti (TiO_6_) was found to be remarkably lower than that without a non-framework Ti zeolite catalyst, and the intermediate of the double-toothed superoxide IM_1b (η^2^) had more reaction activity and stability.

Compared to CTS zeolite, the utilization of PDDA could enrich Ti species on the surface of PTS, which was also accompanied by the presentation of more than two-fold hexahedrally coordinated Ti species (Table 2, d), and it has been proved that the TiO_6_ exhibited two~three-fold higher run activity than that of tetrahedral coordination. PDDA has numerous C_8_H_16_N^+^ chains, which could effectively adsorb Si-OH and Ti-OH, and the Ti-OH exhibited a stronger interaction with PDDA compared with Si-OH [39]. The C_8_H_16_N^+^ chain could not only enrich tetrahedrally coordinated Ti species on the surface but also improve the dispersion.

### 3.2. Formation Mechanism of Short b-Axis-Oriented TS-1 Nanosheets

The TS-1 nanosheets were successfully synthesized with a thickness of 6 nm. PDDA functioned as a mesoporous template and structure-directing agent during the preparation process of PTS. The texture property and chemical state of TS-1 zeolite could be controlled by adjusting the content of PDDA in the precursor gel. The ZSM-5 zeolite with a thinner b-axis was gradually formed in the growth process, and the fluoride anions functioned as a structure-directing agent in the TPA^+^F^−^ ion pair, which conducted the process of microcrystal growth [40]. The abundant cations and anions in PDDA could match with TPA^+^ and material species in the precursor gel.

**Figure 6:**
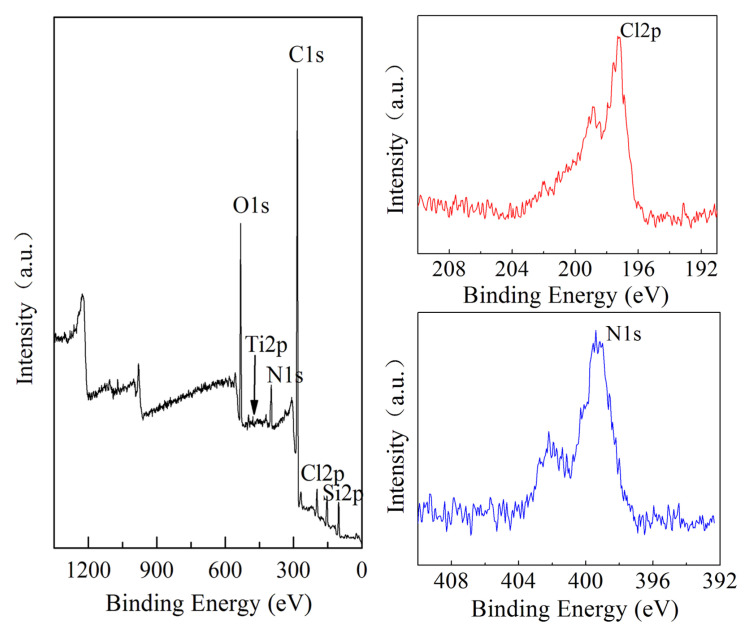
XPS for the precursor of 0.030PTS.

The synthesis progress of nucleation, microcrystals, and nanosheets was investigated. In the initial stage, a network structure was formed between the TPA^+^, silicon, and titanium species, which spontaneously developed into a large number of crystal nuclei. On one hand, the scale of the network structure began to mature and evolve into microcrystals. On the other hand, the [010] plate of microcrystals had the feature of energetically favorable orientation, which was inclined to adsorb additives because of the coordination between Si and O atoms on the [010] plate [41]. The precursor of 0.030PTS was also characterized by XPS, which showed the presence of Cl2p and N1s species on the surface of the precursor (Figure 6).

**Figure 7:**
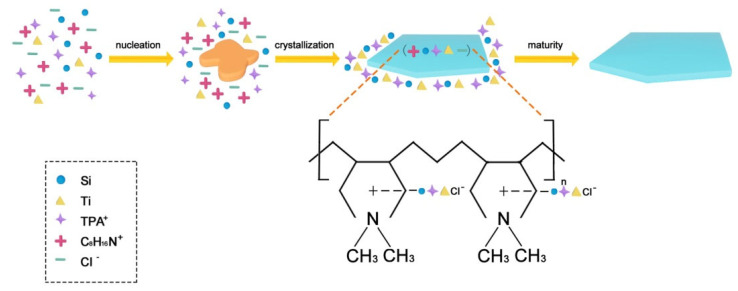
Mechanism of formation of PTS zeolite.

The C_8_H_16_N^+^ and Cl^−^ could be adsorbed on the [010] plate of microcrystals and effectively prescribe the orientation to the TPA^+^, silicon, and titanium species, and the PDDA could occupy the growing orientation along the b-axis. The TPA^+^, silicon, and titanium species were fully dispersed along the a-axis and c-axis and then grew rapidly along the ac crystal plate (Figure 7). Moreover, polymer electrolyte was virtually located between the adjacent ac crystal plates, which could be easily removed through calcination and form a continuous mesoporous channel. Meanwhile, the abundant Ti species were incorporated into the crystal surface as revealed by XPS (Table 2, b,d). Thus, a bulky organic sulfur compound such as DBT with a diameter of 0.887 nm could easily reach the Ti site via continuous mesoporous channels.

### 3.3. Catalytic Performance

**Figure 8:**
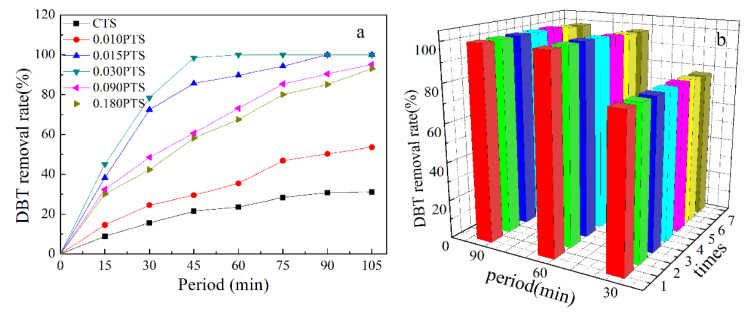
Catalytic test: (**a**) Oxidation of DBT over CTS and stacked nanoplate PTS zeolites. (**b**) Recycle tests for stacked nanosheet 0.030PTS zeolite.

The conventional CTS and PTS zeolites served as the heterogeneous catalysts for the oxidation of a sulfur compound in the model fuel. The PTS zeolites showed superior catalytic activity compared to the CTS zeolite in the oxidation of DBT [42]. The superior catalytic performance was due to the exposed external surface of PTS (105.4–139.5 m^2^/g, Table 1c), which could greatly overcome the restricted access of bulky molecules. Thus, DBT was artificially mixed in noctane and used as a model fuel to evaluate the catalytic performance of the PTS zeolites. Hydrogen peroxide was employed as an oxidizing agent in the reaction. The removal rate of DBT was investigated with a sulfur analyzer (Figure 8a).

The catalytic activity in oxidation was highly related to the diffusion of DBT in the catalyst. The removal rate of DBT could reach 98.5% in 45 min by using 0.030PTS, and the removal rate was 21.5%, 29.5%, 85.6%, 60.7%, and 58.2% corresponding to CTS, 0.010PTS, 0.015PTS, 0.090PTS, and 0.180PTS, respectively. The PDDA could function as a mesoporous template agent for the 0.010PTS zeolite, and the hierarchical intercrystal mesopores were formed after roasting. However, the lowest dosage of PDDA only incorporated a small amount of Ti species onto the surface of the 0.010PTS zeolite (0.79% and 0.29%, Table 2, b,d). As the increment of PDDA, continuous mesopores were formed in 0.015PTS, 0.030PTS, 0.090PTS, and 0.180PTS, and high titanium content was obtained, as shown in Table 2, b,d. It could be concluded that the texture characteristics, surface properties, and phase composition of PTS could be regulated by adjusting the dosage of PDDA. The catalytic activity of zeolite was highly reliant on the accessibility and enrichment degree of the Ti species on the exposed surface.

PDDA that was located between the ac crystal plates (Figure 7) was removed during the calcined process, and the Si and Ti species remained on the zeolite surface. The 0.030PTS zeolite presented the highest desulfurization performance among the prepared TS-1 zeolites because it possessed a higher external area, uniform pore diameter, and moderate surface Ti content. Moreover, the 0.030PTS zeolite could be easily recycled by centrifugation without any pretreatment and the removal rate of DBT was 98.6% in 90 min after seven recycles (Figure 8b). The results demonstrated that the PTS zeolite is a slightly effective and stable catalyst for oxidative desulfurization of DBT and could also be used as a catalyst for other oxidation reactions.

## 4. Conclusions

Herein, we developed a novel and efficient method for preparing TS-1 nanosheets along with short b-axis orientation by utilizing PDDA as a structure-directing agent, which could facilitate the incorporation of Ti species on the [0 1 0] plane of zeolites. The surface Ti content of the prepared TS-1 zeolite could be increased by 59%. The prepared surface of the Ti-rich zeolite catalysts possessed more hexahedrally coordinated Ti species and less anatase, which showed superior catalytic activity in the oxidation desulfurization of DBT compared to CTS zeolite. The PTS zeolites prepared in this study may bring more opportunities for oxidative desulfurization.

## Figures and Tables

**Table 1:** The texture properties of CTS and PTS zeolites as prepared.

Sample	$S_{total}^{a}$ (m^2^/g)	$S_{micro}^{b}$ (m^2^/g)	$S_{external}^{c}$ (m^2^/g)	$V_{total}^{d}$ (cm^−3^/g)	$V_{micro}^{e}$ (cm^−3^/g)	$V_{meso}^{f}$ (cm^−3^/g)	$d_{average}^{g}$ (nm)
CTS	509	423.9	85.11	0.4687	0.1914	0.2773	3.683
0.010PTS	508	368.6	139.5	0.4858	0.1797	0.3061	3.824
0.015PTS	509.3	396.1	113.2	0.3532	0.1825	0.1707	2.774
0.030PTS	504.6	386.8	117.8	0.3220	0.1722	0.1498	2.552
0.090PTS	503.2	397.8	105.4	0.3809	0.1837	0.1972	3.028
0.180PTS	516.3	406	110.3	0.3430	0.1880	0.1550	2.658

^a^ Specific surface area calculated using BET equation. ^b^ Micropore area calculated using t-plot method. ^c^ External surface area. ^d^ Total pore volume at 0.995 of p/p_0_. ^e^ Micropore volume calculated using t-plot method. ^f^ Mesopore volume resulting from total pore volume subtracting micropore volume. ^g^ Average pore diameter calculated by BET specific surface area and total pore volume of cylindrical pore model.

**Table 2:** Surface atomic composition by XPS and Ti relative content by FT-IR.

Sample	${Si}_{Atomic\%}^{a}$	${Ti}_{Atomic\%}^{b}$	${Si/Ti}_{Atomicratio}^{c}$	${Non-Framework\;Ti}_{Atomic\%}^{d}$	$I_{970/800}^{e}$
CTS	31.64	0.69	45.85	0.21	1.18
0.010PTS	30.73	0.79	38.89	0.29	1.09
0.015PTS	30.11	1.00	30.11	0.52	1.14
0.030PTS	31.03	0.95	32.66	0.48	1.10
0.090PTS	30.88	1.10	28.07	0.59	1.13
0.180PTS	28.74	1.08	26.61	0.58	1.12

^a^, ^b^, ^c^, ^d^ Measured by XPS. ^e^ FT-IR intensity ratio of the bands at 970 cm^−1^ and 800 cm^−1^.

## Data Availability

The original contributions presented in the study are included in the article, further inquiries can be directed to the corresponding authors.
